# Epigenetic histone modifications of human transposable elements: genome defense versus exaptation

**DOI:** 10.1186/1759-8753-1-2

**Published:** 2010-01-25

**Authors:** Ahsan Huda, Leonardo Mariño-Ramírez, I King Jordan

**Affiliations:** 1 School of Biology, Georgia Institute of Technology, 310 Ferst Drive, Atlanta, GA 30332, USA; 2 National Center for Biotechnology Information, National Library of Medicine, National Institutes of Health, Bethesda, MD 20894, USA; 3 Computational Biology and Bioinformatics Unit, Biotechnology and Bioindustry Center, Corporacion Colombiana de Investigacion, Agropecuaria - CORPOICA, Km 14 Via a Mosquera, Bogota, Colombia

## Abstract

**Background:**

Transposition is disruptive in nature and, thus, it is imperative for host genomes to evolve mechanisms that suppress the activity of transposable elements (TEs). At the same time, transposition also provides diverse sequences that can be exapted by host genomes as functional elements. These notions form the basis of two competing hypotheses pertaining to the role of epigenetic modifications of TEs in eukaryotic genomes: the genome defense hypothesis and the exaptation hypothesis. To date, all available evidence points to the genome defense hypothesis as the best explanation for the biological role of TE epigenetic modifications.

**Results:**

We evaluated several predictions generated by the genome defense hypothesis versus the exaptation hypothesis using recently characterized epigenetic histone modification data for the human genome. To this end, we mapped chromatin immunoprecipitation sequence tags from 38 histone modifications, characterized in CD4+ T cells, to the human genome and calculated their enrichment and depletion in all families of human TEs. We found that several of these families are significantly enriched or depleted for various histone modifications, both active and repressive. The enrichment of human TE families with active histone modifications is consistent with the exaptation hypothesis and stands in contrast to previous analyses that have found mammalian TEs to be exclusively repressively modified. Comparisons between TE families revealed that older families carry more histone modifications than younger ones, another observation consistent with the exaptation hypothesis. However, data from within family analyses on the relative ages of epigenetically modified elements are consistent with both the genome defense and exaptation hypotheses. Finally, TEs located proximal to genes carry more histone modifications than the ones that are distal to genes, as may be expected if epigenetically modified TEs help to regulate the expression of nearby host genes.

**Conclusions:**

With a few exceptions, most of our findings support the exaptation hypothesis for the role of TE epigenetic modifications when vetted against the genome defense hypothesis. The recruitment of epigenetic modifications may represent an additional mechanism by which TEs can contribute to the regulatory functions of their host genomes.

## Background

Transposable elements (TEs) are mobile DNA sequences that can replicate to extremely high genomic copy numbers. TEs are also widely distributed; they have been found within genomes representing all major eukaryotic lineages. Accordingly, TEs have had a profound impact on the structure, function and evolution of their host genomes. In this study, we explore the relationship between TEs and the epigenetic regulatory mechanisms that are thought to have evolved in response to their proliferation in eukaryotic genomes [1].

Transposition is inherently disruptive in nature. Therefore, in order to ensure their own survival, host genomes must have evolved various repressive mechanisms to guard against deleterious TE insertions. Epigenetic regulatory modifications represent a broad class of silencing mechanisms that may have come into existence in response to the need to repress TEs [1-4]. The notion that epigenetic regulatory systems evolved to silence TEs is known as the 'genome defense hypothesis' [4] and this hypothesis can be taken to make several predictions regarding the epigenetic modifications of TEs. According to the genome defense hypothesis, it be may expected that: (1) younger TEs, that is those that are potentially active, will bear more epigenetic modifications than older inactive TEs; and (2) TEs will bear primarily repressive (gene silencing) modifications rather than active modifications which are associated with gene expression.

An alternative hypothesis to the genome defense model is what we refer to as the 'exaptation hypothesis'. An exaptation describes an organismic feature that currently performs a function for which it was not originally evolved [5]. In the case of TEs, it is well known that a number of formerly selfish or parasitic element sequences have been exapted to provide regulatory and/or coding sequences that serve to increase the fitness of the host [6,7]. For instance, TEs can regulate host genes by serving as the targets of epigenetic histone modifications that spread into adjacent gene loci [2,8]. TE sequences that have been exapted are often anomalously conserved, due to the fact that they are preserved by natural selection after acquiring a function for the host genome [9]. For this reason, exapted TEs tend to be relatively ancient compared to TEs genome-wide.

Consideration of the exaptation hypothesis for TEs in epigenetic terms also yields several specific predictions. According to the TE exaptation model, it is expected that: (1) older and more conserved TEs will bear more epigenetic marks than younger TEs; (2) both active and repressive histone modifications will be targeted to TEs; and (3) TEs closer to genes will bear more histone modifications than more distal TEs.

Our current understanding of the relationship between TEs and epigenetic histone modifications is mainly derived from studies on plants and fungi [10-17]. The vast majority of evidence from these studies points to the genome defense hypothesis as the best explanation for how and why TEs are epigenetically modified. For instance, in *Arabidopsis thaliana*, TE insertions can trigger *de novo *formation of heterochromatin by recruiting repressive histone modifications [2,10]. Similarly, in the yeast *Schizosaccharomyces pombe*, a classical repressive histone tail modification histone H3 lysine 9 trimethylation (H3K9me3) is known to induce the formation of heterochromatin upon a TE insertion [18]. For both plants and yeast, RNA transcripts generated from TEs are thought to trigger an RNA interference related pathway that leads to their epigenetic suppression [13,14].

To date, only a handful of studies have investigated the relationship between mammalian TEs and epigenetic histone modifications. These studies have found that mammalian TEs are targeted primarily by repressive histone tail modifications. The first indication of the involvement of repressive histone modifications with human TEs was unexpectedly discovered by Kondo and Issa in 2003 who found that H3K9me2 is targeted primarily to Alu elements in the human genome [19]. A couple of years later, Martens *et al. *reported varying levels of TE enrichment for repressive marks in repetitive DNA in mouse embryonic stem cells [20]. Recently, a genome-wide map of several histone tail modifications in mouse was published by the Bernstein and Lander groups [8,21]. They found that intracisternal A particle (IAP) and early transposon (ETn) elements were the only families of TEs enriched in repressive histone marks. IAP and ETn are young and active lineages of long terminal repeat (LTR) - retrotransposons and their targeting by repressive modifications is consistent with the host's need to suppress their activity. Another recent study in the mouse by the Jenuwein group also found an enrichment of the repressive mark H3K27me3 in silent genes and nearby short interspersed nuclear elements (SINEs) [22]. Thus, the majority of evidence to date points to the genome defense hypothesis as the best explanation for the role of epigenetic modifications targeted to mammalian TE sequences.

Recently, a series of chromatin immunoprecipitation followed by high-throughput sequencing (ChIP-Seq) experiments have been performed by the Keji Zhao group, which together yield a genome-wide map of histone tail modifications in human CD4^+ ^T cells [23,24]. These data provide a unique opportunity to qualitatively and quantitatively investigate the relationship between epigenetic histone modifications and human TEs, and to test the predictions of the genome defense hypothesis versus the exaptation hypothesis.

## Results and discussion

### Characterization of TE histone modifications

Previously, a series of ChIP-Seq analyses were used to determine the genome-wide distributions of 38 histone tail modifications in human CD4^+ ^T cells [23,24]. For these studies, sequence tags corresponding to specifically modified histones were characterized using the Illumina-Solexa platform and the tags were mapped to the human genome sequence using the software provided by the vendor. This approach only yields unambiguously mapped sequence tags that correspond to unique genomic locations. In other words, all tags that map to repetitive sequences are eliminated from consideration. Since we are analysing TEs here, many of which are repetitive DNA sequences, we used our own mapping procedure (see Methods) to recover many of the sequence tags that map to more than one location in the genome and therefore had been discarded in the previous studies.

Our tag-to-genome mapping procedure yielded a total of 369,225,759 mapped sequence tags over the 38 histone modifications. This figure represents an increase of 144,125,239 tags (64%) over the previously employed mapping procedure, for an average increase of 3,792,769 tags per modification. Differences in the numbers of mapped tags for each histone modification can be seen in Additional file 1, Figure S1. For human TE sequences, we mapped an additional 77,065,760 tags over the 38 modifications.

The genome defense hypothesis for TE epigenetic modifications predicts that TEs will bear primarily repressive, rather than active, histone tail modifications, whereas the exaptation hypothesis holds that both active and repressive histone modifications will be targeted to TEs. The histone tail modifications analysed here were characterized as active or repressive based on their enrichment in genes with different CD4^+ ^T cell expression levels using a previously described approach [24]. To apply this approach, we established presence/absence calls for each modification in the promoter regions of human genes by comparing promoter modification tag counts to corresponding genomic background tag counts as described in the Methods. We then calculated the fold enrichment of expression by comparing the average CD4^+ ^T cell expression level of genes marked as present for a particular modification with the average expression level of genes that do not display any enrichment of the same modification (Additional file 1, Figure S2). There are 28 histone tail modifications characterized as active using this approach and 10 modifications characterized as repressive. This method reveals the effects of individual histone modifications on gene expression, presumably based on how they help to determine open versus closed chromatin states. In other words, active modifications are associated with the active expression of human gene sequences, whereas repressive modifications are associated with gene silencing. Accordingly, the genome defense hypothesis would predict the targeting of potentially active TEs with repressive histone tail modifications.

**Figure 1:**
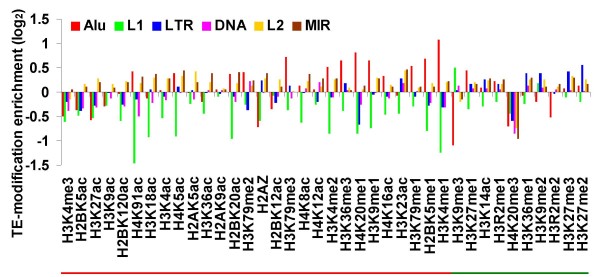
**Enrichment or depletion of 38 individual histone modifications in transposable element (TE) families**. Log2 normalized ratio of the number of tags of each of the 38 histone modifications located within each TE family over the total number of tags taken as the genomic background is shown. Statistical significance determined by the G test (see Additional file 1, Table S1).

A variety of TEs are found in the human genome [25]. Retrotransposons constitute the vast majority of these sequences with Alu and L1 being the youngest and most abundant families and MIR and L2 being older inactive lineages of SINEs and LINEs, respectively. LTR retrotransposons are a less abundant but more diverse group of retrotransposons, with very few extant subfamilies. DNA-type elements make up a distinct class of TEs, which are substantially less abundant than retrotransposons in the human genome. We evaluated the relative enrichment of each histone tail modification over six classes (families) of human TEs: Alu, L1, LTR, DNA, L2 and MIR (Figure 1). To do this, a fold-change approach similar to that used to characterize active versus repressive modifications was used. For each histone tail modification, the TE family-specific tag counts were compared against the genomic background for that modification (Methods). Thus, the fold-change values represent the extent to which TE families are enriched or depleted for each of the 38 histone tail modifications. This generated a total of 228 (6 × 38) TE-by-modification fold-change values, all of which were statistically significant (Additional file 1, Table S1; G test 0 = *P *< 2.1e-5). TE epigenetic histone modifications vary widely according to the TE family, as well as the identity of the specific modification. There are numerous active and repressive modifications that are enriched for different TE families. Some families, such as Alu and L2, appear to be enriched for active modifications, whereas others, such as L1 and LTR, are depleted for active modifications and/or enriched for repressive modifications. Cleary, human TE sequences are bound by histones that are subject to numerous active and repressive epigenetic modifications.

Human TEs are distributed non-randomly across the genome with respect to gene locations and guanine-cytosine (GC) content. For instance, Alu elements are enriched in and around genes in high GC rich regions of the genome, whereas L1 elements are found primarily in AT rich DNA in intergenic regions [25]. Thus, using the entire genomic background of histone modification tag counts to compute the modification enrichments for TE families with distinct genomic distributions could bias the results. In order to control for this possibility, we re-calculated the enrichment of histone modifications by comparing the histone modification tag counts of each TE to a background tag count computed from a genomic window encompassing that TE (Methods). This local approach to computing TE histone modification enrichments does not qualitatively change the results obtained when compared to the global approach. Indeed, the TE-histone modification enrichment ratios computed using global versus local histone modification background tag counts are highly correlated (0.91 = *r *= 0.99) for each of the six classes (families) of TEs evaluated (Additional file 1, Figure S3). For comparison, the relative enrichments of TE-histone tail modifications calculated in this way are shown in Additional file 1, Figure S4. Whether the TE-histone modification enrichments are computed using global or local modification tag counts, human TEs show evidence of being targeted by a number of different active and repressive epigenetic marks.

### Active versus repressive TE histone modifications

**Figure 2:**
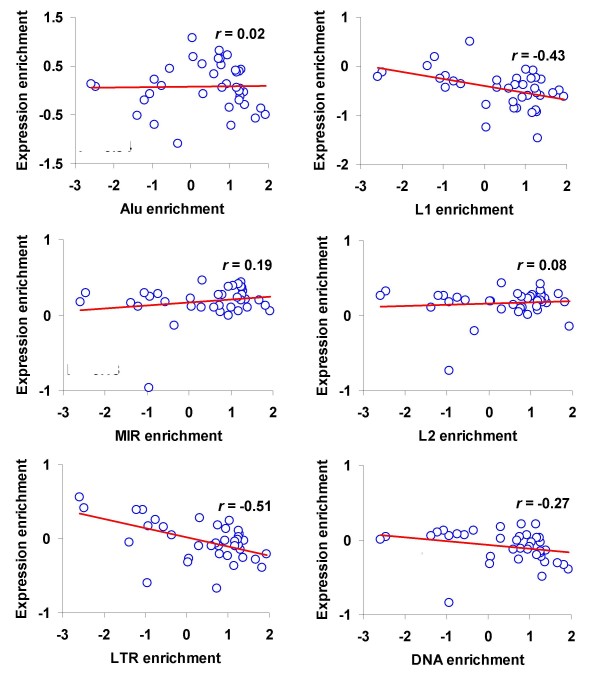
**Correlation between enrichment of histone modifications in transposable element (TE) families and for human gene expression**. The enrichment of 38 histone modifications in human gene expression (Additional file 1, Figure S2) is plotted against the same in six TE families (Figure 1). See Methods for details and Additional file 1, Table S2 for statistical significance. Pearson correlation coefficient values (r) are shown.

The genome defense hypothesis for TE epigenetic modifications predicts that TEs that are capable of transposition will be targeted by repressive histone modifications in order to suppress their activity. The exaptation hypothesis, on the other hand, predicts that older and more conserved TEs will bear more epigenetic marks. These older TEs will have lost the ability to transpose and are more likely to have been exapted to play some role for their host genome. To distinguish between these models, we correlated the histone tail modification enrichment for specific TE families with the histone tail modification gene expression enrichment values. The genome defense hypothesis would predict a negative correlation since repressive modifications should target actively expressing TEs with the potential to transpose, whereas the exaptation model may predict a positive correlation or no correlation at all. None of the TE families shows a statistically significant relationship between TE and gene expression enrichment for individual histone modifications (Figure 2 and Additional file 1, Table S2). The same analysis was done using the local approach to computing the histone modification background tag counts, as described in the previous section, and the results are qualitatively similar when this technique is applied (Additional file 1, Figure S5). These results are not consistent with the genome defense hypothesis, but it is unclear whether they reflect the absence of genome defense, exaptation or some combination thereof.

**Figure 3:**
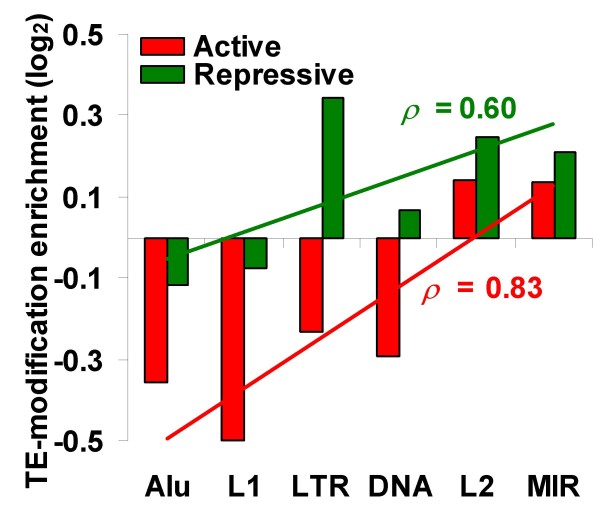
**Enrichment or depletion of active and repressive histone modifications in retrotransposons**. Histone modifications were classified as active or repressive based on expression enrichment (Additional file 1, Figure S2). The log2 normalized ratios of the number of tags of active or repressive modifications located within each family of retrotransposons over the total number of tags taken as the genomic background is shown. Retrotransposon families are arranged according to their relative ages. Spearman rank correlations (ρ) between active and repressive transposable element (TE)-modification enrichments (depletions) and the relative ages of TE families are shown.

To further evaluate the active versus repressive TE modification predictions for the genome defense versus exaptation hypotheses, we grouped and summed the histone tail modification tags into the 28 active and 10 repressive modifications. The enrichment of active and repressive modifications was calculated by co-locating the tags from each class with TE sequences from each family and comparing the TE family-specific active or repressive tag counts with the genomic background. The data shows considerable variation between active and repressive modification enrichments in different lineages of TEs (Figure 3). Alus and L1s are significantly depleted in both active and repressive modifications, with relatively fewer active modifications. LTR elements show depletion for active modifications and enrichment for repressive modifications, which is entirely consistent with the predictions of the genome defense model. On the other hand, L2 and mammalian-wide interspersed repeat (MIR) elements show enrichment for both active and repressive modifications consistent with the exaptation model.

The data on active versus repressive histone modifications for TE families also bears on the predictions relating epigenetic modifications to the ages of TEs. The genome defense hypothesis predicts that potentially active younger TEs will bear more epigenetic modifications than older TEs, while the exaptation model predicts that more ancient conserved TEs will bear more epigenetic modifications. The different families of TEs shown in Figure 3 have different relative ages, on average, with Alu elements being the youngest and MIRs being the oldest [young-to-old: Alu-L1-LTR-DNA-L2-MIR] [25]. The enrichments of both active and repressive modifications are positively correlated with the age of the TE families (Figure 3); in other words, older families of elements tend to be more modified than younger families. The same analysis was done using the local approach to computing the histone modification background tag counts, as described in the previous section, and the results are qualitatively similar when this technique is applied (Additional file 1, Figure S6). These data are consistent with the exaptation hypothesis for TE modifications, as opposed to the genome defense model, and suggest that many older TE sequences may be preserved, at least in part, due to the contributions they make the epigenetic environment of the human genome.

### TE ages and histone modifications

The divergence of an individual TE insertion from its subfamily consensus sequence is a barometer of the time elapsed since its insertion and is, thus, a good measure for its relative age [25]. As shown in Figure 3, a comparison between TE families indicates a positive correlation between element ages and the extent of histone tail modifications. This observation is consistent with the exaptation hypothesis, which predicts that older TEs will bear more epigenetic modifications. However, these results may be confounded by comparisons between families made up of very different kinds of TEs with distinct insertion mechanisms, genomic distributions and life histories. In order to evaluate the relationship between element ages and histone tail modifications in a more controlled way, we compared the extent of TE histone modifications with the relative ages of TE insertions within the Alu and L1 families of elements. The Alu and L1 families were chosen for two reasons: first, they are numerous and abundant providing statistical resolution on the question; secondly, and more importantly, they have well-characterized subfamilies the relative ages of which are known [25-27]. The relative ages of individual Alu and L1 insertions can be inferred by comparing their sequences to the consensus sequences of their subfamilies (Additional file 1, Figures S11 and S12) and these data are provided in the output of the RepeatMasker program used to annotate the elements. We computed the average element-to-subfamily consensus sequence divergence for all Alu and L1 subfamilies and compared these values to the extent of active and repressive histone modifications that map to members of the individual subfamilies.

**Figure 4:**
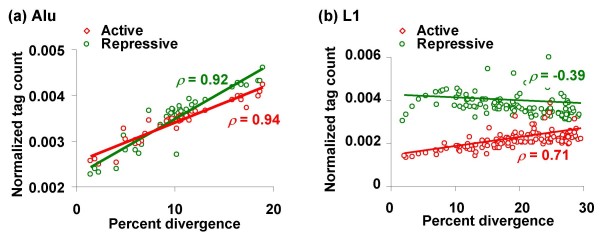
**Age of Alu and L1 elements versus their histone modifications**. Relative ages of Alu (a) and L1 (b) subfamilies, as determined by divergence from subfamily consensus sequences, are plotted against their respective tag counts normalized by genomic length. Spearman rank correlations (ρ) between tag counts and percent divergence are shown for active (red) and repressive (green) modifications separately (significance values are in Additional file 1, Table S4).

The within-family analyses of the relationship between the relative ages of Alu elements and their histone modifications yield results that are most consistent with the exaptation hypothesis (Figure 4a). Alu element ages are significantly positively correlated with both active (*ρ *= 0.94, *P *= 4e-20) and repressive (*ρ *= 0.92, *P *= 9e-18) histone modifications (Additional file 1, Table S4). These data indicate that members of older Alu subfamilies are subject to more active and repressive modifications, which stands in contrast to the prediction of the genome defense model that younger elements should be more repressed.

The relationships between the ages of L1 elements and their histone modification states appear to support both the genome defense and exaptation models (Figure 4b). The ages of L1 elements are negatively correlated with repressive modifications (*ρ *= -0.39, *P *= 5e-6) and positively correlated with active modifications (*ρ *= 0.71, *P *= 4e-20) (Additional file 1, Table S4). The relative abundance of repressive modifications of younger L1s is consistent with the genome defense model, whereas the data for the increasing active modifications of older L1 elements are consistent with the exaptation model. Taken together, the within-family data for Alu and L1 elements display a complex view of the relationship between TE ages and histone modifications suggesting interplay between the genome defense and exaptation hypotheses.

### TE-gene locations and histone modifications

**Figure 5:**
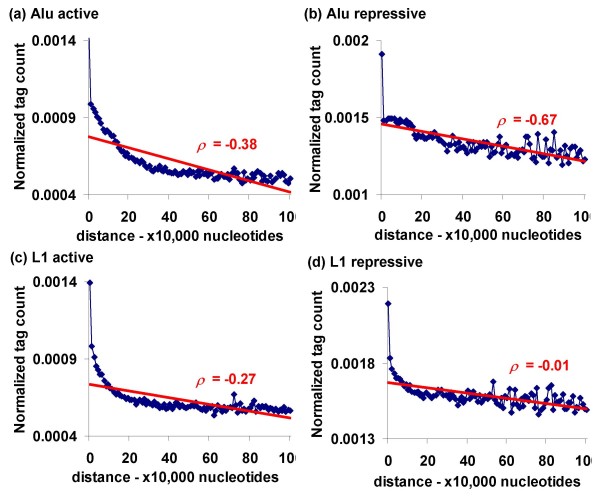
**Transposable element (TE) distance from genes versus histone modifications**. Distances between Alu (a and b) and L1 (c and d) sequences and the nearest genes are binned in 10 kb bins and plotted against the number of active (a and c) or repressive (b and d) histone modification tags mapped to the TE sequences normalized by their lengths. Spearman rank correlations (ρ) are shown and significance values are in Additional file 1, Table S3.

The exaptation hypothesis predicts that TEs proximal to host genes would bear more histone modifications than those that are distal to genes, since these modifications are more likely to effect the regulation of the genes. In order to test this prediction, we analysed the Alu and L1 TE families and associated every TE sequence to the nearest gene. The corresponding tag counts of active and repressive histone modifications in TEs were binned according to their distance from genes. Only uniquely mapped TE-tags that could be assigned unambiguous genomic locations were used for this analysis. Alu and L1 were chosen both for their genomic abundance and for the fact that they have distinct genomic distributions: Alus are enriched near genes, whereas L1s are found more often in intergenic regions. For both Alu and L1, we observed negative correlations (Alu active *ρ *= -0.38, *P *= 5e-5, Alu repressive *ρ *= -0.67, *P *= 9e-14, L1 active *ρ *= -0.27, *P *= 0.003, L1 repressive *ρ *= -0.01, *P *= 0.46) between TE insertion distances from genes and histone modifications (Figure 5 and Additional file 1, Table S3). Moreover, TEs that lie within gene boundaries are modified at much higher levels compared to those outside of genes. These findings are in agreement with the exaptation hypothesis. The same analysis was done using both unique and repetitively mapping tags, and the results are qualitatively unchanged when this more comprehensive approach is taken (Additional file 1, Figure S7).

## Conclusions

### Comparison with previous results

While most work to date on mammalian histone modifications has focused on non-repetitive DNA, there have been four recent studies on the histone modification status of mammalian repetitive sequence elements, three in mouse [8,20,22] and one in human [19]. The previous studies focused on repressive histone modifications and they turned up a number of cases where mammalian TEs, including SINEs, LTR and DNA elements, were found to be enriched for specific histone modifications. We compare the results of these previous studies with the findings reported here in Table 1. Interestingly, the results reported here agree and disagree with those of previous studies in equal measure. When specific histone modifications are considered for individual TE classes, there are six cases where histone modifications previously identified to be enriched for a given TE class are enriched in the same class in our study, and there are six cases where previously enriched TE-modifications are found to be depleted here. These discrepancies underscore the extent to which histone modifications, particularly those that target TEs, may be cell-type specific, since the different studies that are being compared analysed different cell types. Indeed, the study of Martens *et al. *evaluated multiple cell types and found that histone modifications of TEs were more variable across cell types than those of tandem satellite repeats [20]. This was attributed to the fact that tandemly repeated DNA, such as that found around centromeres, form more stable chromatin architectural elements and tandem repeats are present in more constitutively heterochromatic environments. Interspersed repeats, on the other hand, may be more prone to cell-type specific *in situ *formation of heterochromatic regions dispersed among the euchromatic portion of the genome. This has been seen in plants where insertions of TEs lead to the localized spread of repressive chromatin [2]. In any case, a deeper understanding of how human TEs are epigenetically modified, along with the regulatory implications, will require a comparison of TE-modifications across a variety of cell types.

**Table 1:** Comparison of transposable element (TE) histone modification enrichments found in this study with those of previous studies.

**Enriched in previous study**^a^	**Status in current study**^b^
Kondo and Issa 2003 (Human) [19]	

SINE: H3K9me2	Depleted

Martens *et al. *2005 (Mouse) [20]	

SINE: H3K9me3	Depleted

SINE: H3K27me3	Enriched

SINE: H4K20me3	Depleted

LTR: H3K9me3	Enriched

LTR: H3K27me3	Enriched

LTR: H4K20me3	Depleted

DNA: H3K27me3	Enriched

DNA: H4K20me3	Depleted

Mikkelsen *et al. *2007 (Mouse) [8]	

LTR: H3K9me3	Enriched

LTR: H4K20me3	Depleted

Pauler *et al. *2008 (Mouse) [22]	

SINE: H3K27me3	Enriched

^a ^TE classes (SINE, LINE, LTR or DNA) that were shown to be enriched for specific histone modifications (as shown) in previous studies.

^b ^Status of the same TE class-histone modification pairs as enriched or depleted in this study

### Exaptation as a local or global phenomenon

Exaptation refers to the evolutionary process whereby an organismic feature comes to play some role for which it was not originally evolved or selected [5]. TEs are primarily selfish genetic elements that evolved solely virtue of their ability to transpose and thus out-replicate the host genomes in which they reside [28,29]. They do not owe their evolutionary success to any ability to provide functional utility to their hosts. However, at this time it is widely recognized that a number of individual TE sequences have been exapted to play some positive role for their host genomes [6,7]. Exaptation of individual TE sequences may include cases where TEs become incorporated into host protein coding genes or cases where TEs provide regulatory sequences that help to control the expression of host genes. Such examples of TE exaptation are very much in keeping with the original definition of exaptation as referring to a series of individual, and largely contingent, cases. However, the genome-scale approach taken here to exploring the implications of TE epigenetic modifications entails the consideration of exaptation as a more global, rather than a strictly local, phenomenon. This is because there are particular features of TEs, specifically their ability to recruit epigenetic modifications, which are shared across many elements over the entire genome and which, in turn, allow individual insertions to be exapted. This does not mean that all TEs in the genome are exapted. Rather, the data reported here suggest that there are genome-scale signals, in terms of how the TEs are epigenetically modified, which indicate an overall potential for individual human TE sequences to be exapted. Consideration of exaptation as a global or genome-scale phenomenon as it relates to TEs reveals how inherent features of the elements, such as their ability to be transcribed or their dispersed repetitive nature, serve to recruit the very epigenetic machinery that will allow them to affect the regulation of nearby genes. Having established this global pattern of TE epigenetic exaptation, further inquiry can now be used to identify individual cases of interest. We give specific examples of how individual cases of TE epigenetic exaptation may be uncovered in the following section.

### Caveats and future directions

As mentioned previously, TE epigenetic modifications are certain to be cell-type specific to some extent. Here, we only analysed histone modifications of human TEs in a single cell type - CD4^+ ^T cells. As more and more genome-scale histone modification data sets become available, it will become possible to systematically evaluate changes in the histone modification states of TEs across tissues. This is particularly relevant for a deeper interrogation of the genome defense hypothesis. Vertical transmission (inheritance) of novel TE insertions, along with their mutagenic effects, is dependant upon transposition events that occur in the germline, as opposed to TE insertions in somatic tissue, which is an evolutionary dead end. For this reason, one may expect that the most vigorous genome defense mechanisms would be employed in germline tissue. Thus, it is possible that the predictions of the genome defense model, which are not supported for the most part in this study, may be borne out if germline tissue was evaluated in the same way as done here for somatic tissue. However, there is some evidence that suggests this may not be the case for human TEs. Alu elements, which make up a huge fraction of the methylated DNA in the human genome in somatic tissues, are actually hypomethylated in the male germline [30]. This may represent an evolutionary strategy for the elements, whereby the TEs mitigate their deleterious effects in somatic tissue by reducing transposition therein and yet allow for the transmission of new insertions across generations by relaxing element suppression in the germline [31]. This kind of strategy can be seen for P elements in Drosophila, which utilize alternative splicing to encode a repressor protein in somatic tissue and a transposase in the germline [32]. Nevertheless, a better understanding of the role epigenetic histone modifications in the repression of heritable TE insertions will require the analysis of germline tissue.

The genome-wide mapping of 38 histone modifications in the human genome enabled us to thoroughly investigate the relationship between TEs and epigenetic histone modifications. We tested several predictions generated by two competing hypotheses - the genome defense hypothesis and the exaptation hypothesis - in the light of epigenetic histone modifications. Consistent with the exaptation hypothesis, we found that the overall enrichment of histone modifications is positively correlated with the increasing age of TE insertions, and TEs proximal to human genes bear more histone marks than TEs distal to genes. We also found support for the genome defense hypothesis for certain cases, but the majority of our data and analyses support the exaptation hypothesis.

Thus, for the human genome, some epigenetic modifications of TEs may serve to regulate the expression of host genes rather than to silence the elements themselves. More definitive proof of epigenetically related exaptation of TEs will require the analysis of individual cases whereby specific TE sequences have been exapted to regulate host genes. These could include TE-derived promoter sequences, which provide local regulatory sequences and transcription start sites to host genes, and/or TE-derived enhancers that regulate genes from more distal locations. An evaluation of how such TE-derived regulatory sequences are epigenetically modified across different cell types along with an examination of how cell-type specific modifications correspond to expression differences should help to reveal epigenetic routes by which TEs influence their host genomes.

## Methods

### Tag-to-genome mapping

The genome-wide distributions of 38 histone tail modifications were previously evaluated in human CD4^+ ^T cells using ChIP-Seq with the Illumina-Solexa platform [23,24]. The mapping protocol used in these studies did not allow for the consideration of histone modifications at repetitive DNA sequences, since they removed redundantly mapping sequence tags. Therefore, we employed a heuristic mapping procedure for the data generated in these ChIP-Seq studies in order to be able to analyse sequence tags that map to repetitive DNA. To do this, we downloaded 140 sequence tag libraries corresponding to the 38 previously characterized CD4^+ ^T cell histone tail modifications from the NCBI Short Read Archive (SRP000200 and SRP000201) [33]. Sequence reads and their respective quality scores were converted from Illumina-Solexa format to the standard (Sanger) fastq format, and the MAQ (Mapping and Alignment with Qualities) program was used to map each fastq library to the March 2006 human genome reference sequence (NCBI Build 36.1, hg18 assembly). MAQ uses a mapping algorithm that utilizes the tag sequences along with their quality scores to determine the highest scoring match to the genomic location [34]. MAQ was run in such a way that tags with more than one identically scoring best tag-to-genome alignment, *i.e. *repetitively mapping tags, were randomly assigned to one genomic location. This procedure allowed us to avoid the elimination of sequence tags that have high scoring tag-to-genome alignments but map to more than one location. Since human TEs can be characterized into related groups (classes, families and subfamilies), using this heuristic mapping procedure provides an unambiguous way to evaluate differences in the frequencies of specific histone modifications between related groups of TEs.

### Gene expression-histone modification enrichment analysis

We downloaded the Refseq annotations of experimentally characterized transcription start sites from the database of transcription start sites (DBTSS) [35,36], and mapped them to the human genome reference sequence (hg18) at the UCSC Genome Browser [37]. CD4^+ ^T cell expression data corresponding to the mapped Refseq genes were taken from the Novartis Gene Expression Atlas 2 [38]. We were able to obtain unambiguously mapped transcription start sites and gene expression data for 12,644 human genes. We defined promoter regions as 1000 nucleotides upstream and 200 nucleotides downstream of the transcription start sites. We located the number of tags corresponding to each histone tail modifications in each promoter region. The number of tags of each modification in a promoter region was converted to a binary presence/absence call using a genomic background tag distribution and a conservative threshold determined by the Poisson distribution and incorporating Bonferroni correction for multiple tests [24].

Combing the CD4^+ ^T cell gene expression data with promoter histone modification presence/absence calls, we calculated the expression enrichment for each histone modification using the following formula:

In addition, for each histone tail promoter modification, the significance of the difference in average CD4^+ ^T cell gene expression levels for genes with and without the modification was evaluated using the Student's *t*-test.

### TE-histone modification enrichment analysis

We downloaded RepeatMasker [39] annotations (version 3.2.7) of TE locations for the human genome reference sequence (hg18) from the UCSC genome browser. Using the TE genomic coordinates and our tag-to-genome mapping data, we co-located the tags that correspond to each histone tail modification with TE sequences in the human genome. In this way, we obtained the number of tags of each histone tail modification that map to TE sequences in the human genome.

The TE-histone modification mapping dataset was divided into six classes (families) of TEs [40,41] which are: Alu, MIR, L1, L2, DNA transposons and LTR-retrotransposons. We normalized the number of histone modification tags in each class (family) of TE sequences by the total genomic length of these TE sequences in the class (family), and compared the normalized TE tag counts to either (1) genome-wide background tag counts or (2) locally computed genomic background tag counts. Genome-wide background tag counts are the total number of tags for each modification divided by the length of the genome. To obtain local histone modification background tag counts for TE classes (families), for each individual TE insertion, a background tag count was computed by randomly sampling a non-TE sequence of the same size from within a 1 megabase genomic window surrounding that TE. These individual local background tag counts were then averaged over all TE insertions of a given class (family). The following formulas were used for enrichment calculations:

where

### Statistical analyses

The statistical significance of TE-histone modification enrichment values were calculated using the goodness of fit *G*-test, which uses a log-likelihood ratio comparing the observed to expected tag counts. The *P*-value thresholds for the *G*-tests were adjusted using the Bonferroni correction for multiple tests. Prior to correlation analysis, all data distributions were checked for normality using Q-Q plots to visually compare the observed distributions against theoretical normal distributions (Additional file 1, Figures S8-S10). Data with distributions that were deemed to be normal were correlated using Pearson correlation (*r*) and data with distributions that were deemed to be non-normal were correlated using Spearman rank correlation (*ρ*). Note that when data are binned, such as for the distance from gene computation, correlations are calculated on the unbinned data. Statistical significance values for correlations were computed using an approximation to the Student's *t*-distribution with *n*-2 degrees of freedom [42].

## Abbreviations

ChIP-Seq: chromatin immunoprecipitation followed by high-throughput sequencing; ETn: early transposon; GC: guanine-cystosine; IAP: intracisternal A particle; LTR: long terminal repeat; MIR: mammalian-wide interspersed repeat; SINE: short interspersed nuclear element; TE: transposable elements.

## Competing interests

The authors declare that they have no competing interests.

## Authors' contributions

IKJ and AH conceived of and designed the study, performed computational analyses and wrote up the results. LMR provided technical expertise and assistance for dataset acquisition, curation and analysis. All authors read and approved the manuscript.

## Supplementary Material

Additional file 1 **Supplementary material**. Figures S1-12 and Tables S1-4 are included in the supplementary material file. Click here for file
