# Supplementary material for: Epigenetic histone modifications of human transposable elements: genome defense versus exaptation
Source: Mob DNA. 2010 Jan 25;1:2. doi: 10.1186/1759-8753-1-2 (PMC2836006; doi:10.1186/1759-8753-1-2)
Supplement: Additional file 1 — Supplementary material. Figures S1-12 and Tables S1-4 are included in the supplementary material file. [file 1759-8753-1-2-S1.PPT]

## Slide 1
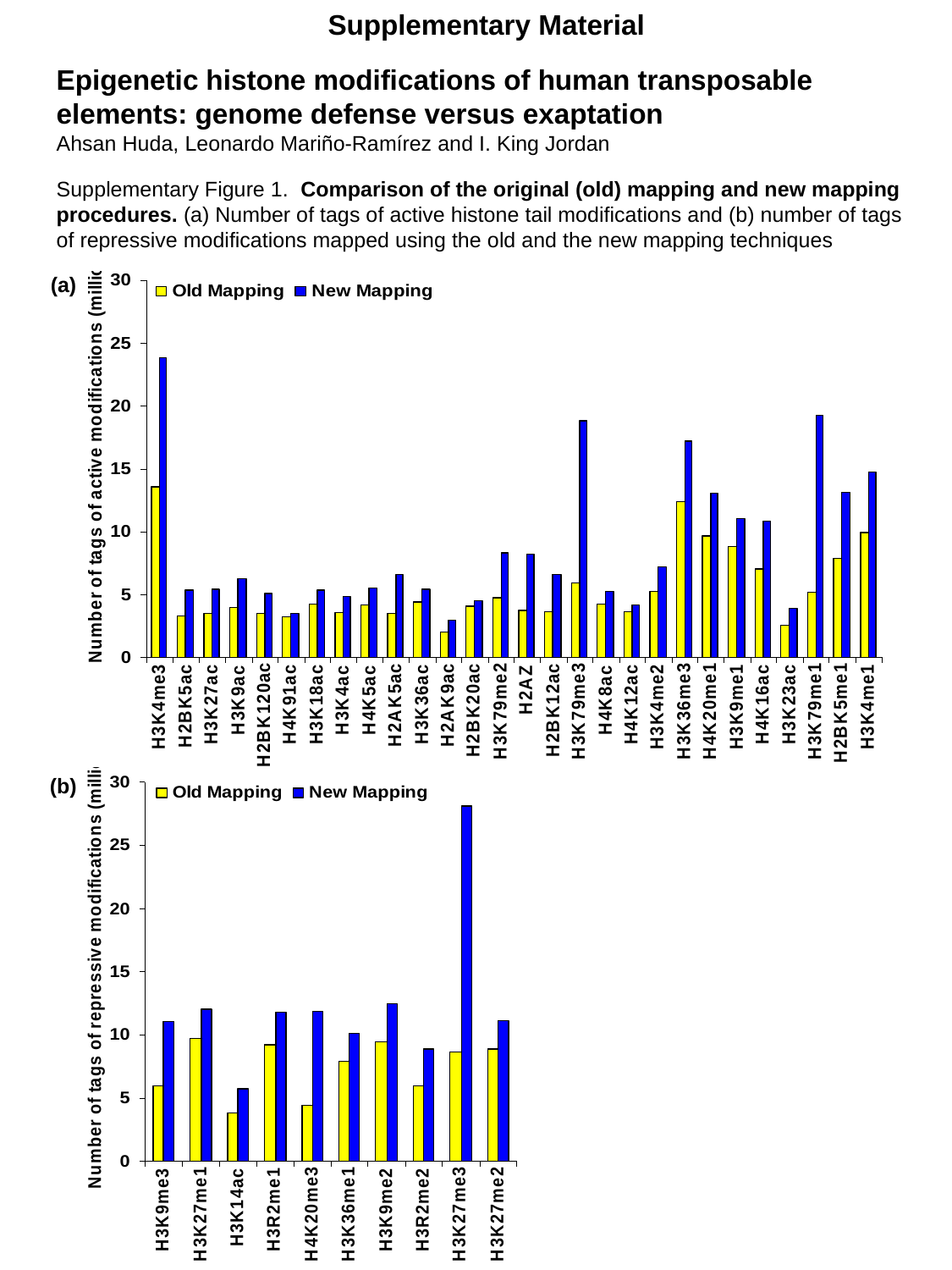

Supplementary Material
Epigenetic histone modifications of human transposable
elements: genome defense versus exaptation
Ahsan Huda, Leonardo Mariño-Ramírez and I. King Jordan
Supplementary Figure 1. Comparison of the original (old) mapping and new mapping
procedures. (a) Number of tags of active histone tail modifications and (b) number of tags
of repressive modifications mapped using the old and the new mapping techniques
(a)
(b)

## Slide 2
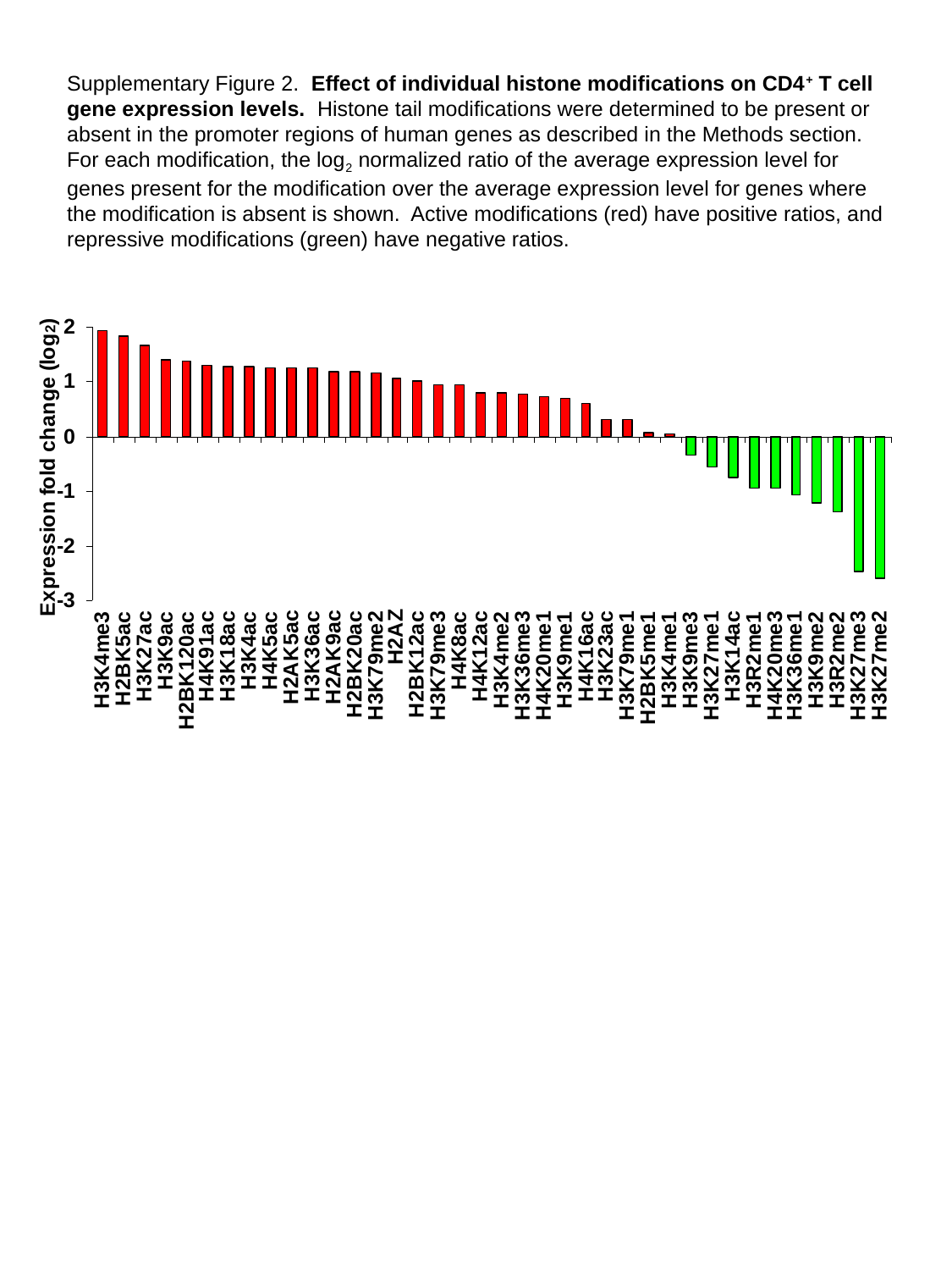

Supplementary Figure 2. Effect of individual histone modifications on CD4+ T cell
gene expression levels. Histone tail modifications were determined to be present or
absent in the promoter regions of human genes as described in the Methods section.
For each modification, the log2 normalized ratio of the average expression level for
genes present for the modification over the average expression level for genes where
the modification is absent is shown. Active modifications (red) have positive ratios, and
repressive modifications (green) have negative ratios.

## Slide 3
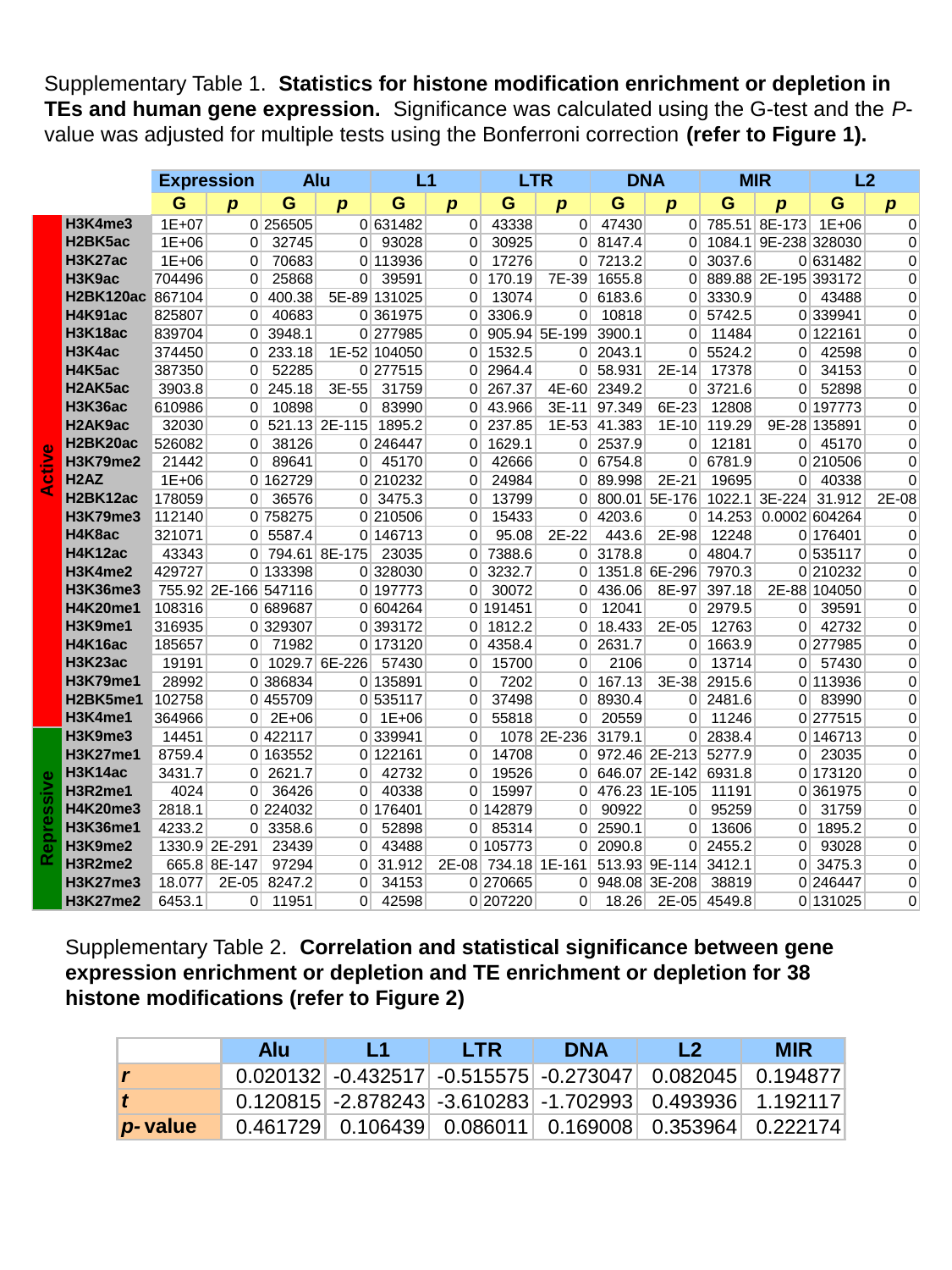

Supplementary Table 1. Statistics for histone modification enrichment or depletion in TEs and human gene expression. Significance was calculated using the G-test and the P-value was adjusted for multiple tests using the Bonferroni correction (refer to Figure 1).
Supplementary Table 2. Correlation and statistical significance between gene expression enrichment or depletion and TE enrichment or depletion for 38 histone modifications (refer to Figure 2)

## Slide 4
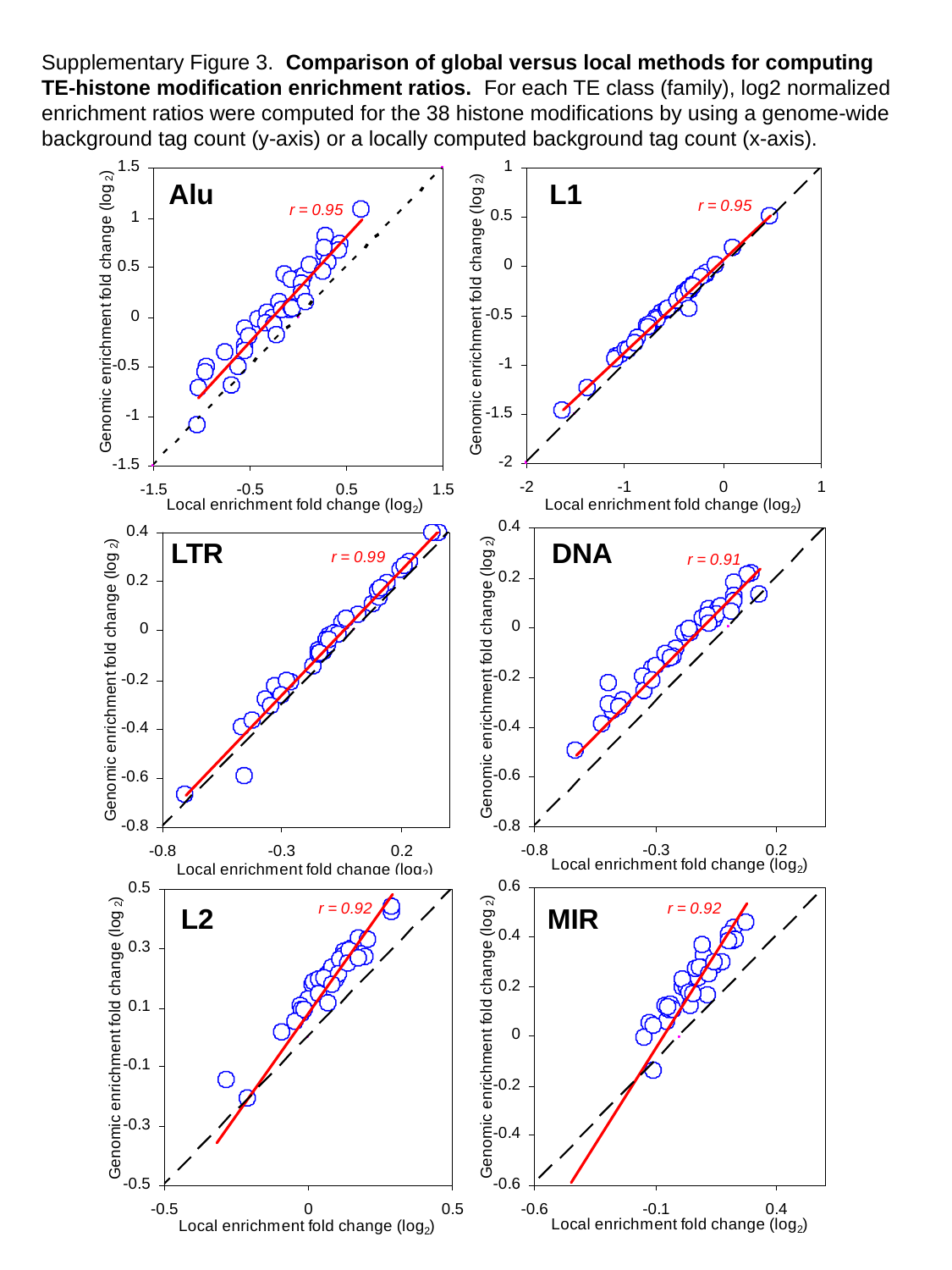

Supplementary Figure 3. Comparison of global versus local methods for computing
TE-histone modification enrichment ratios. For each TE class (family), log2 normalized
enrichment ratios were computed for the 38 histone modifications by using a genome-wide
background tag count (y-axis) or a locally computed background tag count (x-axis).
Alu			L1
LTR			DNA
L2		 MIR

## Slide 5
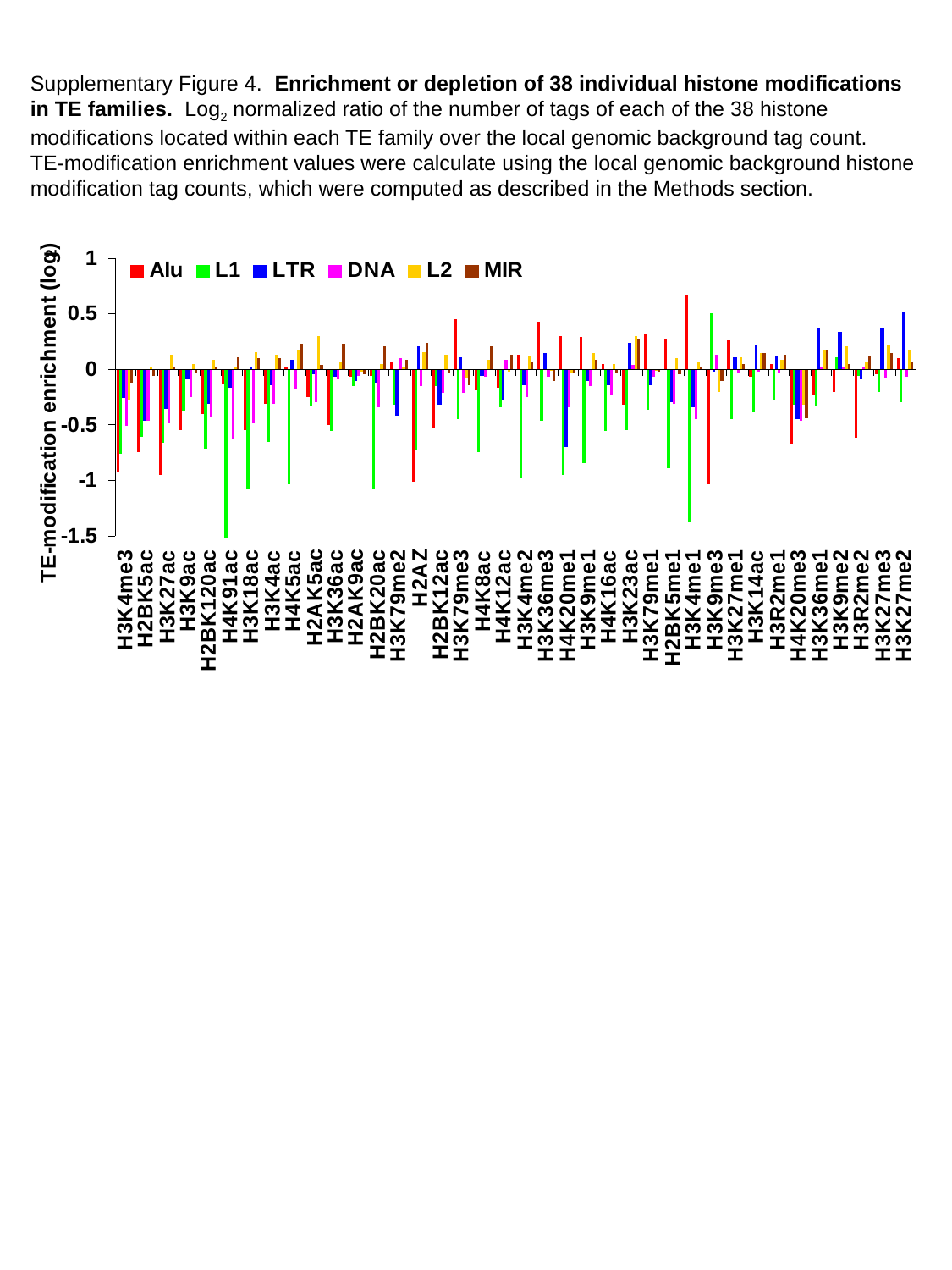

Supplementary Figure 4. Enrichment or depletion of 38 individual histone modifications
in TE families. Log2 normalized ratio of the number of tags of each of the 38 histone
modifications located within each TE family over the local genomic background tag count.
TE-modification enrichment values were calculate using the local genomic background histone
modification tag counts, which were computed as described in the Methods section.

## Slide 6
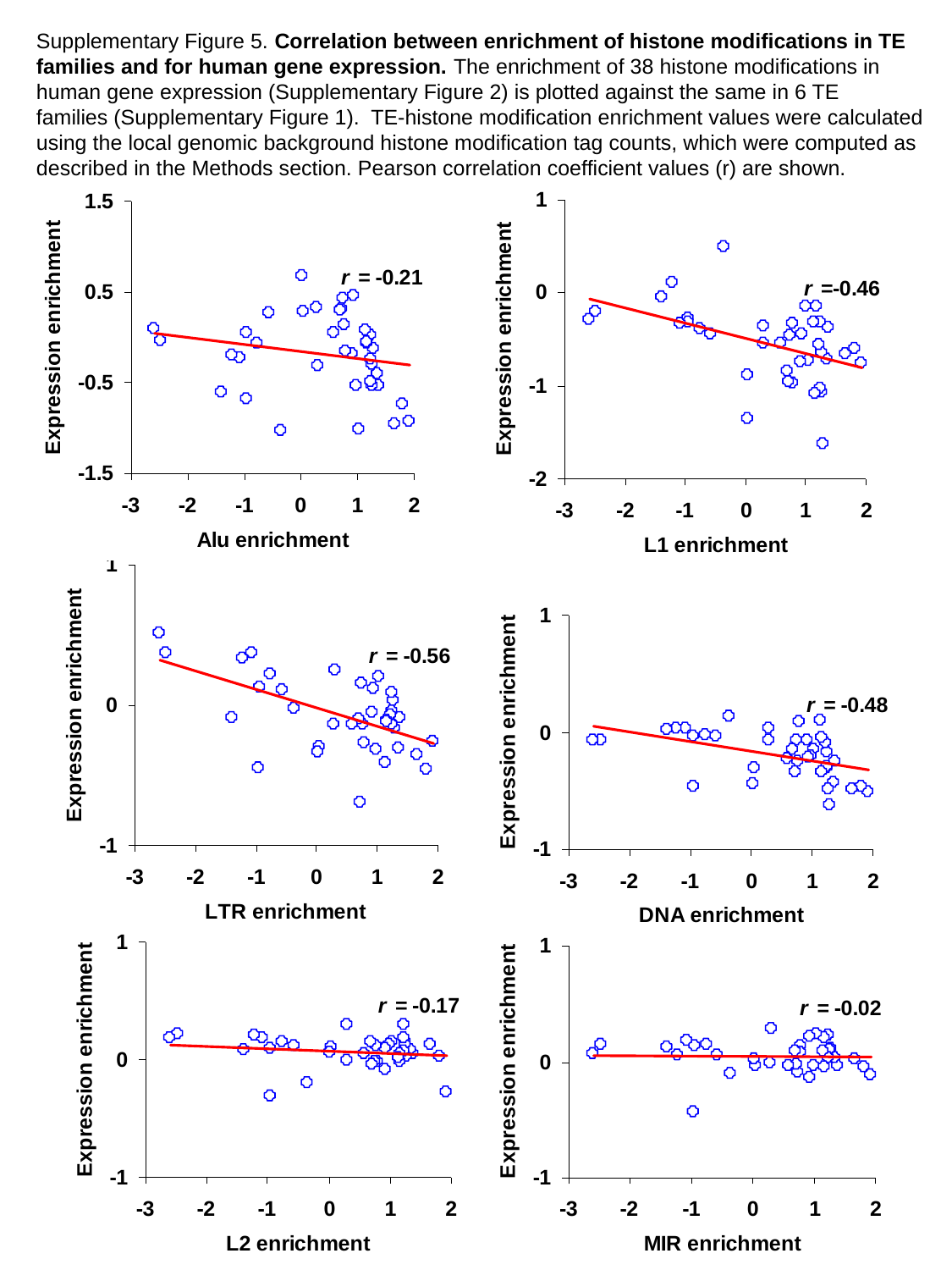

Supplementary Figure 5. Correlation between enrichment of histone modifications in TE
families and for human gene expression. The enrichment of 38 histone modifications in
human gene expression (Supplementary Figure 2) is plotted against the same in 6 TE
families (Supplementary Figure 1). TE-histone modification enrichment values were calculated
using the local genomic background histone modification tag counts, which were computed as
described in the Methods section. Pearson correlation coefficient values (r) are shown.

## Slide 7
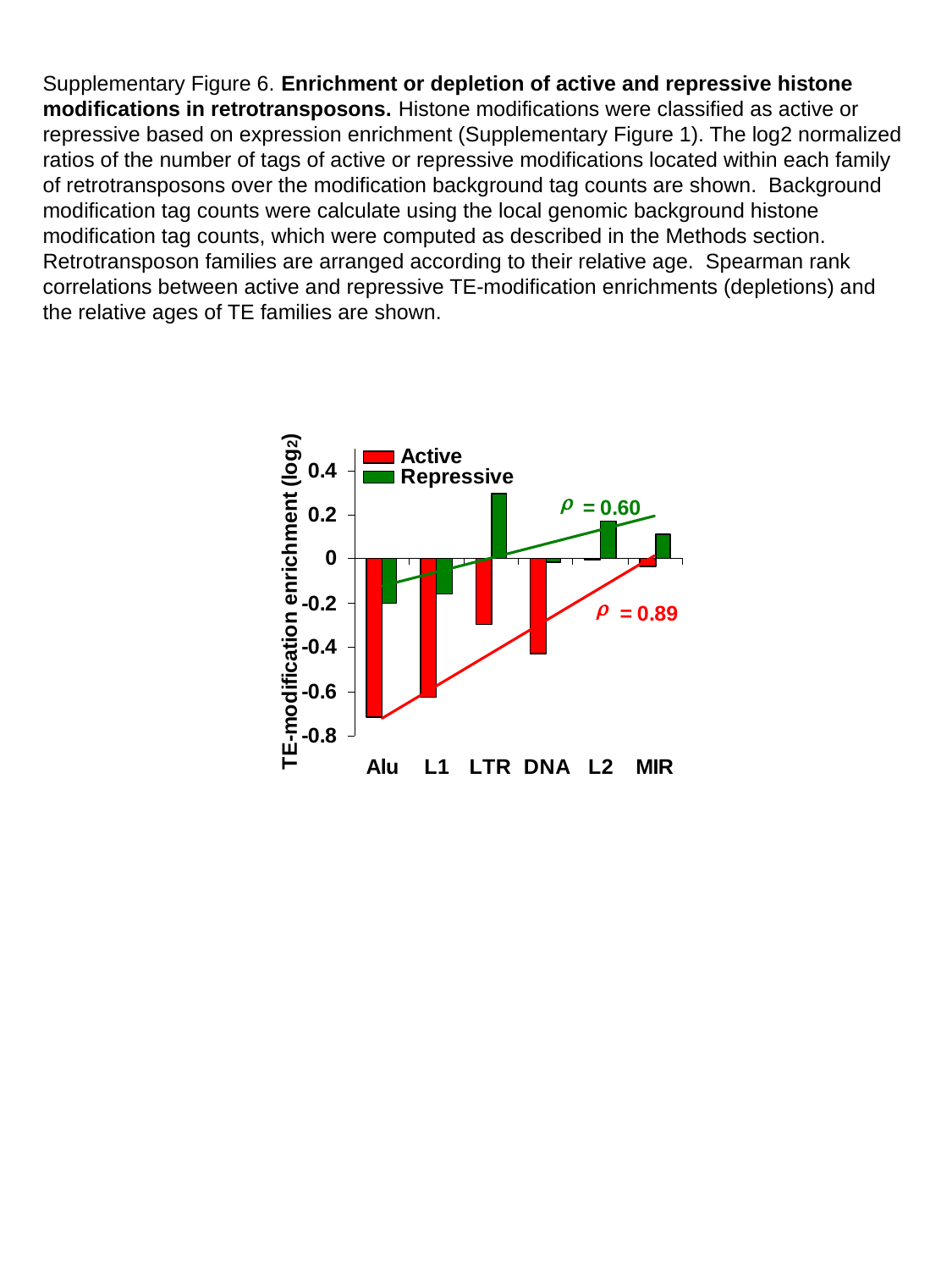

Supplementary Figure 6. Enrichment or depletion of active and repressive histone
modifications in retrotransposons. Histone modifications were classified as active or
repressive based on expression enrichment (Supplementary Figure 1). The log2 normalized
ratios of the number of tags of active or repressive modifications located within each family
of retrotransposons over the modification background tag counts are shown. Background
modification tag counts were calculate using the local genomic background histone
modification tag counts, which were computed as described in the Methods section.
Retrotransposon families are arranged according to their relative age. Spearman rank
correlations between active and repressive TE-modification enrichments (depletions) and
the relative ages of TE families are shown.

## Slide 8
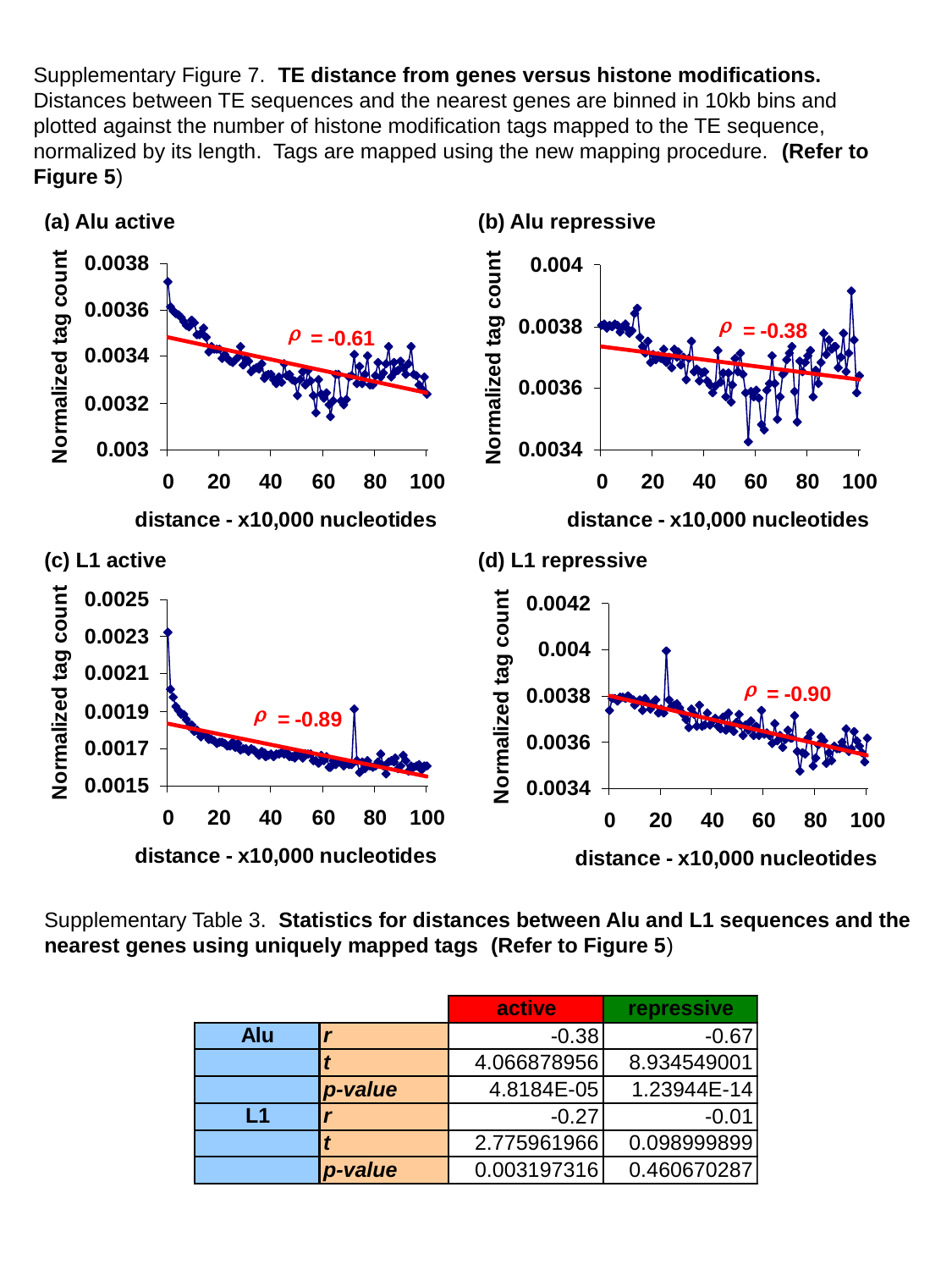

Supplementary Figure 7. TE distance from genes versus histone modifications. Distances between TE sequences and the nearest genes are binned in 10kb bins and plotted against the number of histone modification tags mapped to the TE sequence, normalized by its length. Tags are mapped using the new mapping procedure. (Refer to Figure 5)
(a) Alu active		 (b) Alu repressive
(c) L1 active			 (d) L1 repressive
Supplementary Table 3. Statistics for distances between Alu and L1 sequences and the nearest genes using uniquely mapped tags (Refer to Figure 5)

## Slide 9
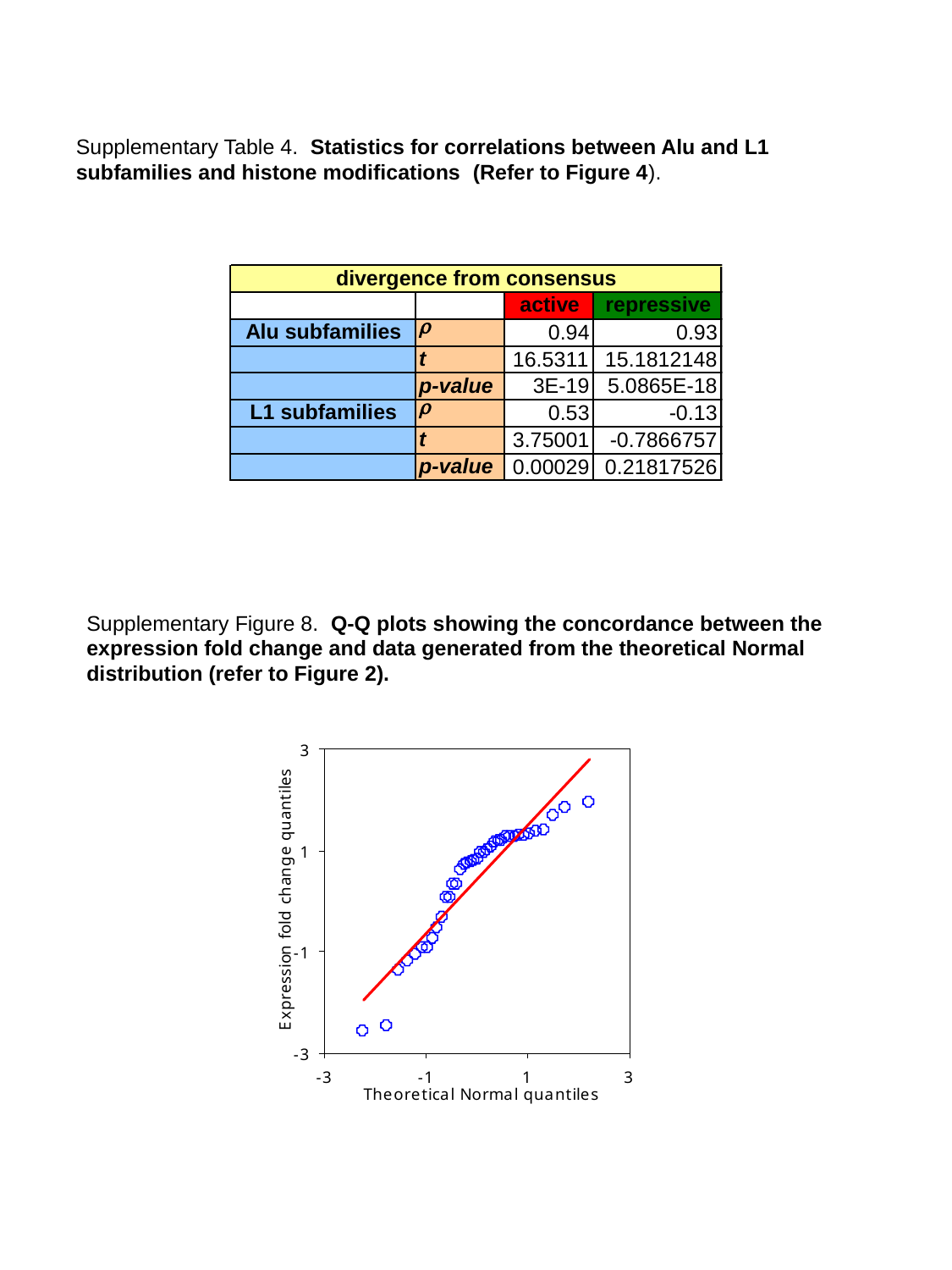

Supplementary Table 4. Statistics for correlations between Alu and L1 subfamilies and histone modifications (Refer to Figure 4).
Supplementary Figure 8. Q-Q plots showing the concordance between the expression fold change and data generated from the theoretical Normal distribution (refer to Figure 2).

## Slide 10
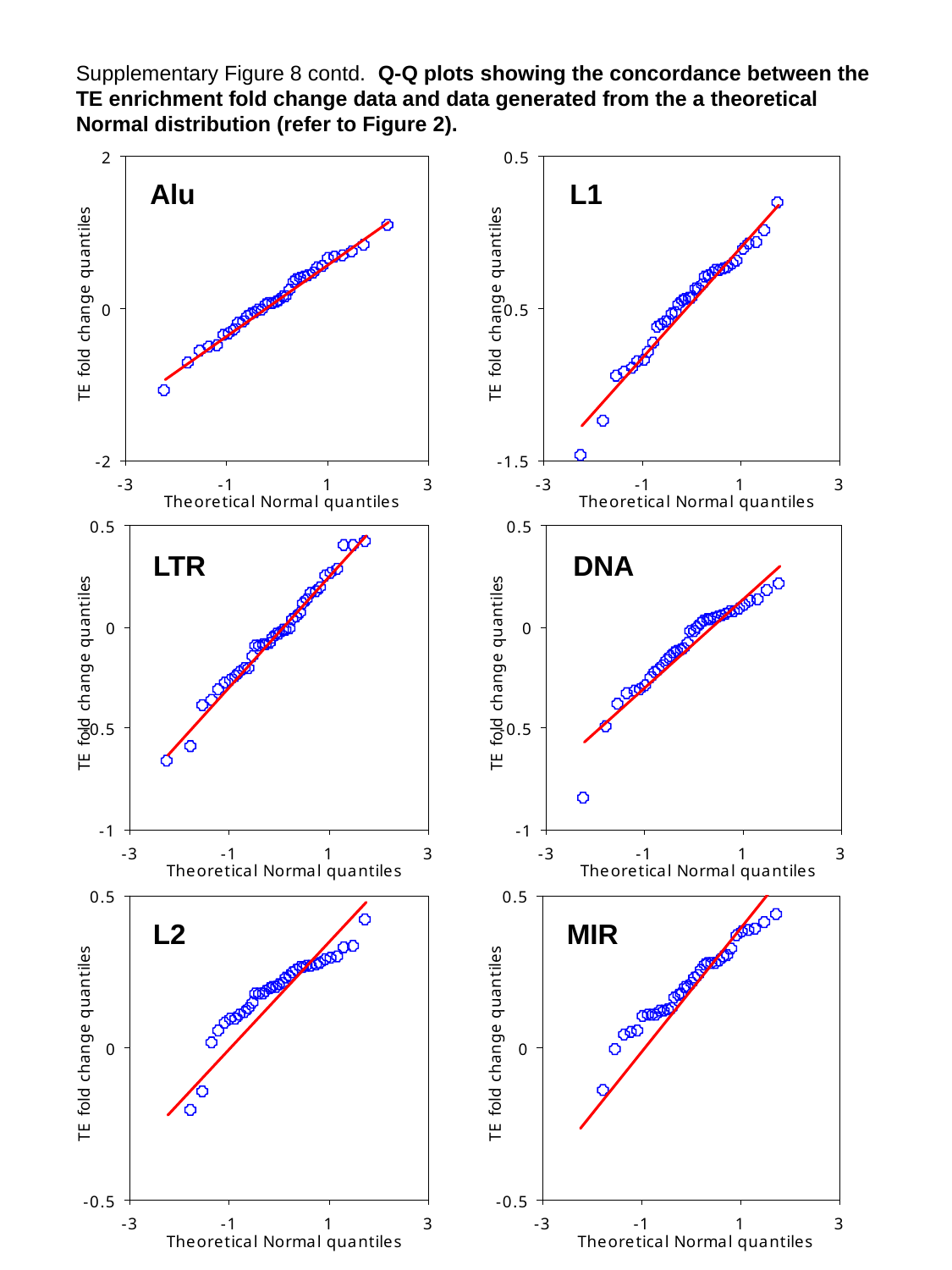

Supplementary Figure 8 contd. Q-Q plots showing the concordance between the TE enrichment fold change data and data generated from the a theoretical Normal distribution (refer to Figure 2).
Alu			 L1
LTR		 	 DNA
L2		 MIR

## Slide 11
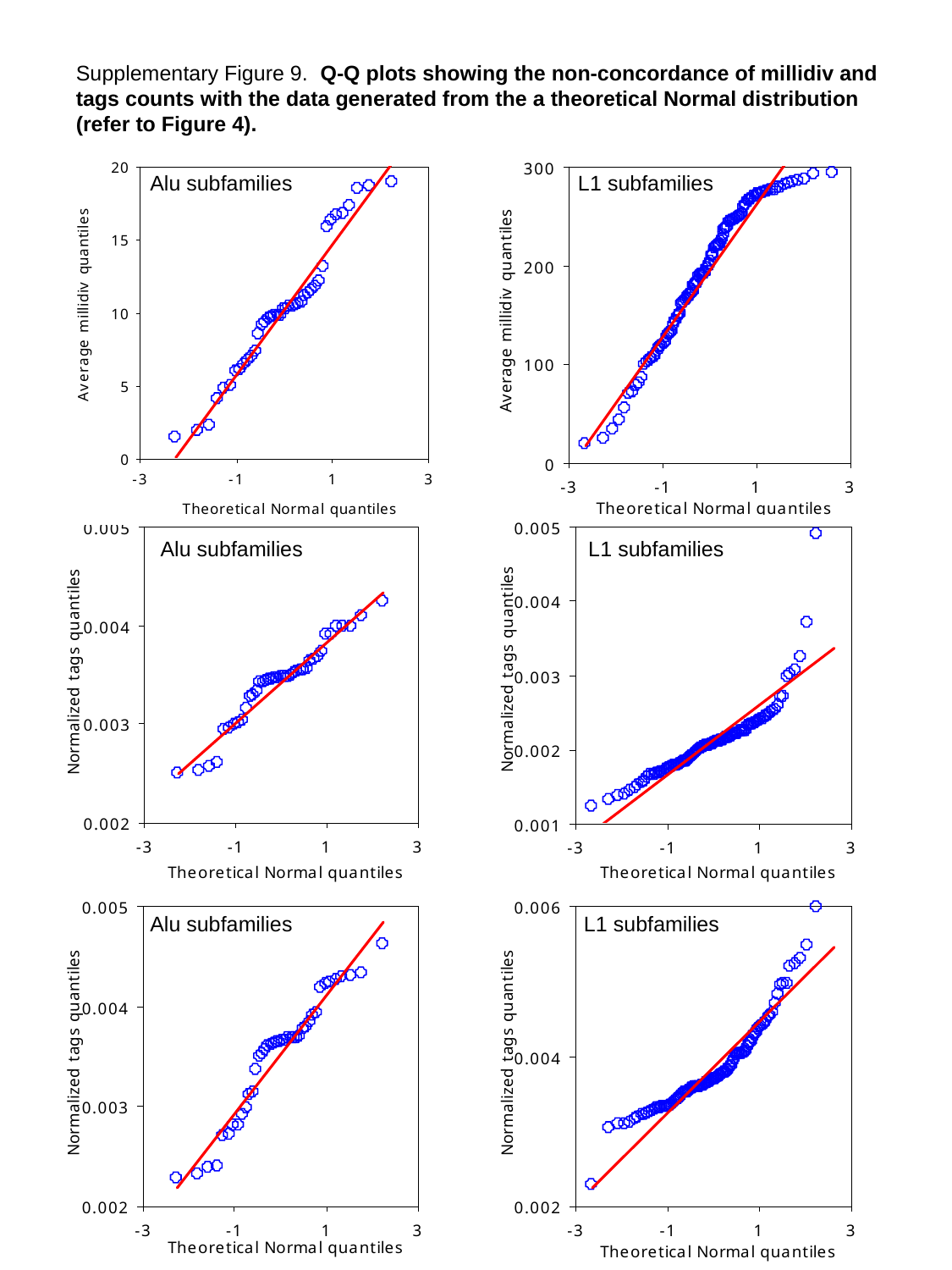

Supplementary Figure 9. Q-Q plots showing the non-concordance of millidiv and tags counts with the data generated from the a theoretical Normal distribution (refer to Figure 4).
Alu subfamilies		 L1 subfamilies
Alu subfamilies		 L1 subfamilies
Alu subfamilies		 L1 subfamilies

## Slide 12
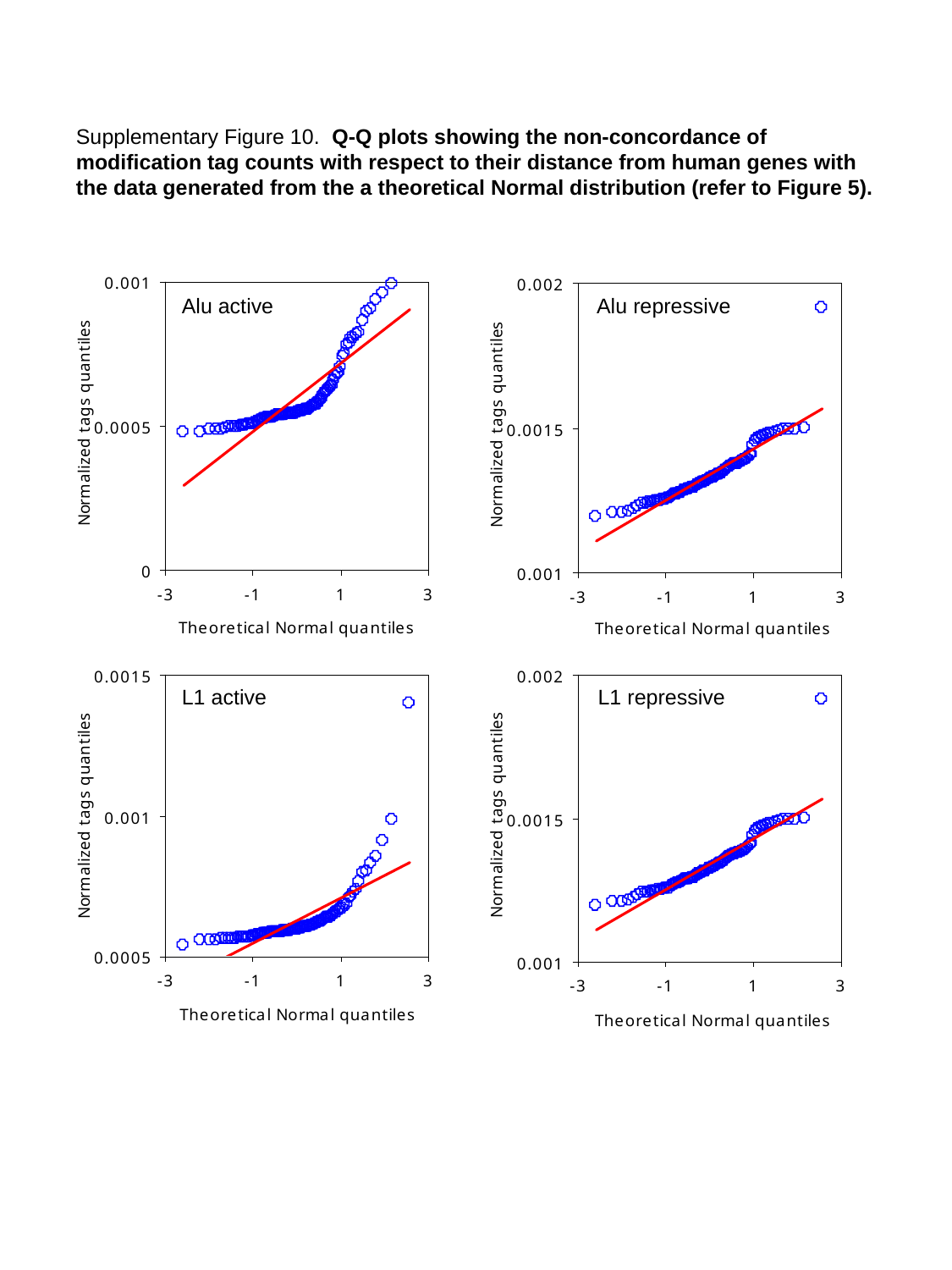

Supplementary Figure 10. Q-Q plots showing the non-concordance of modification tag counts with respect to their distance from human genes with the data generated from the a theoretical Normal distribution (refer to Figure 5).
Alu active		 	 Alu repressive
L1 active		 	 L1 repressive

## Slide 13
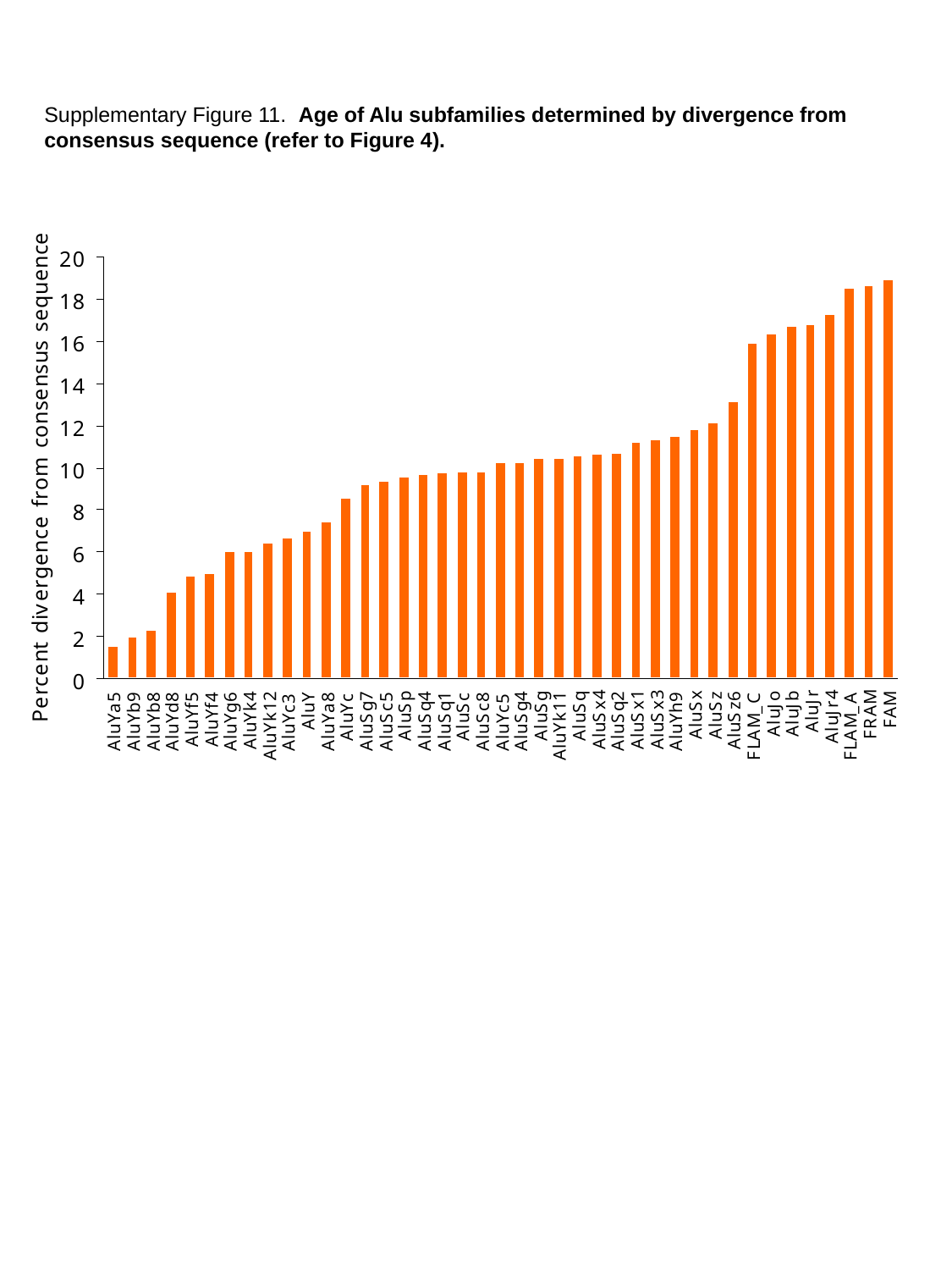

Supplementary Figure 11. Age of Alu subfamilies determined by divergence from consensus sequence (refer to Figure 4).

## Slide 14
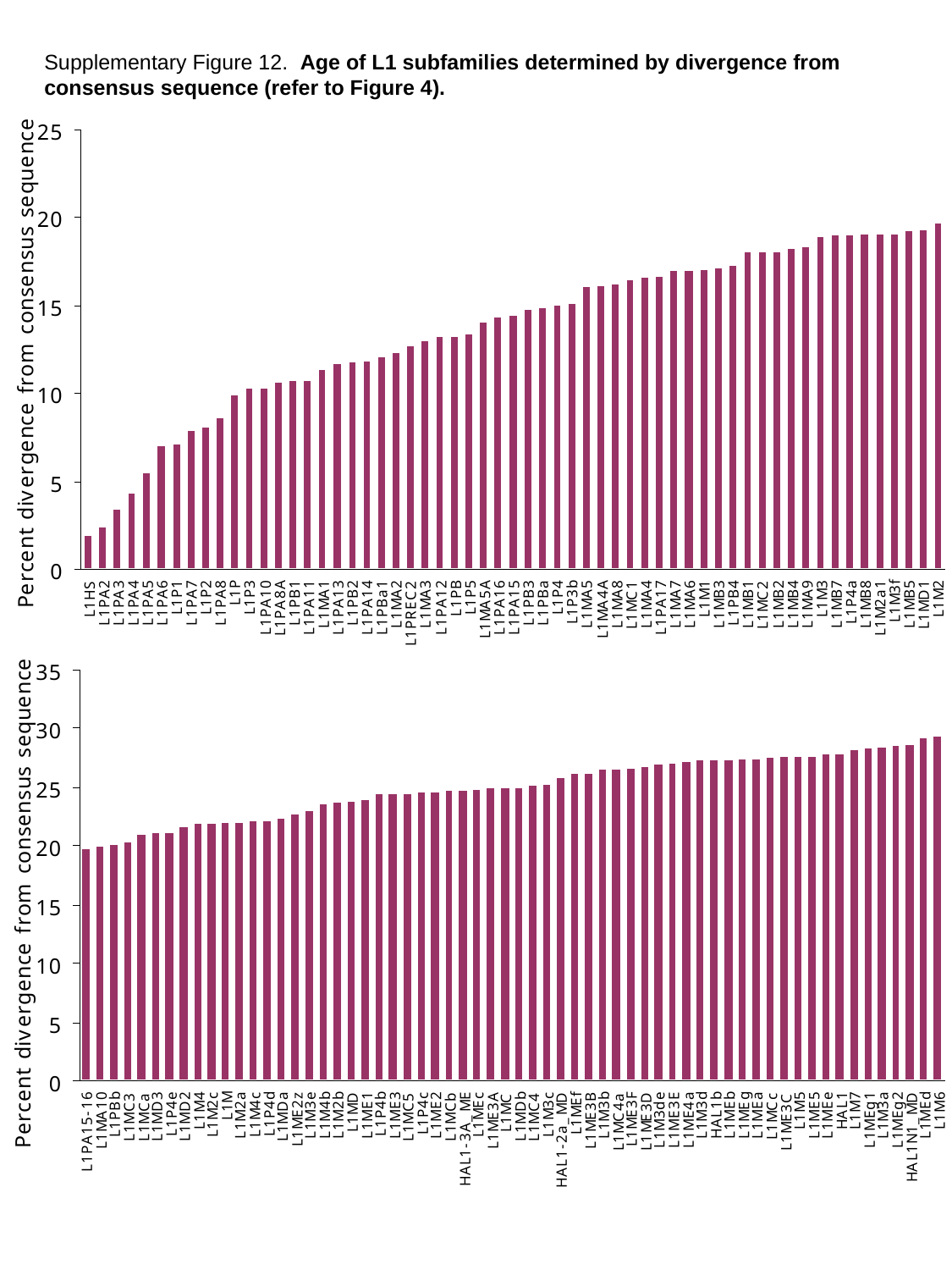

Supplementary Figure 12. Age of L1 subfamilies determined by divergence from consensus sequence (refer to Figure 4).
